# Exocytosis of Nanoparticles: A Comprehensive Review

**DOI:** 10.3390/nano13152215

**Published:** 2023-07-30

**Authors:** Jie Liu, Yuan-Yuan Liu, Chen-Si Li, Aoneng Cao, Haifang Wang

**Affiliations:** Institute of Nanochemistry and Nanobiology, Shanghai University, Shanghai 200444, China

**Keywords:** exocytosis, nanomaterials, lysosomal, Golgi, kinetics, nanosafety

## Abstract

Both biomedical applications and safety assessments of manufactured nanomaterials require a thorough understanding of the interaction between nanomaterials and cells, including how nanomaterials enter cells, transport within cells, and leave cells. However, compared to the extensively studied uptake and trafficking of nanoparticles (NPs) in cells, less attention has been paid to the exocytosis of NPs. Yet exocytosis is an indispensable process of regulating the content of NPs in cells, which in turn influences, even decides, the toxicity of NPs to cells. A comprehensive understanding of the mechanisms and influencing factors of the exocytosis of NPs is not only essential for the safety assessment of NPs but also helpful for guiding the design of safe and highly effective NP-based materials for various purposes. Herein, we review the current status and progress of studies on the exocytosis of NPs. Firstly, we introduce experimental procedures and considerations. Then, exocytosis mechanisms/pathways are summarized with a detailed introduction of the main pathways (lysosomal and endoplasmic reticulum/Golgi pathway) and the role of microtubules; the patterns of exocytosis kinetics are presented and discussed. Subsequently, the influencing factors (initial content and location of intracellular NPs, physiochemical properties of NPs, cell type, and extracellular conditions) are fully discussed. Although there are inconsistent results, some rules are obtained, like smaller and charged NPs are more easily excreted. Finally, the challenges and future directions in the field have been discussed.

## 1. Introduction

More and more nanomaterials have been developed and intended for in vivo applications, such as disease diagnosis and treatment with improved sensitivity and efficiency in biomedicine, taking advantage of the unique physical and chemical properties of nanomaterials [1,2]. At the same time, even more nanomaterials not intended for in vivo applications may also eventually enter human bodies, and thus the safety assessment of nanomaterials has long been an important issue. Both biomedical applications and safety assessments of nanomaterials require a deep understanding of their behavior in organisms. The first and fundamental step is to understand how nanoparticles (NPs) enter cells, translocate in cells, and leave cells.

The uptake and trafficking of various NPs in cells have been studied extensively and have gained a deep understanding, including the aspects of intracellular uptake, endocytosis pathways, organelle localization, and related influencing factors [3,4,5]. Compared with the investigations on endocytosis, less attention has been paid to the exocytosis of NPs, and sometimes the results obtained are inconsistent in many aspects [3,6,7]. Current exocytosis studies focus on a few NPs such as SiO_2_ NPs [8,9,10], Au NPs [11,12], quantum dots (QDs) [13,14,15], and polymers [16,17]. Unfortunately, most of the time, the exocytosis of NPs is simply measured as a supplement to the cellular uptake and transport of NPs in cells [18], or even worse, some researchers believe that the exocytosis of NPs is trivial or negligible [19].

In fact, exocytosis is as important as endocytosis in the interaction of nanomaterials and cells [17,20]. It is the process of discharging unwanted cargo and other macromolecules from cells [21,22], a process opposite to the endocytosis of NPs [23]. Understanding the exocytosis of NPs means to answer how and how fast intracellular NPs move out of cells and to what extent intracellular NPs can still remain in the cells. The endocytosis and exocytosis of NPs jointly decide the content of NPs in cells, while the retention of NPs in cells is indirectly and directly related to their cytotoxicity [11,24,25,26,27]. NPs trapped in cells for a long period may induce various adverse effects, such as cell growth inhibition, asymmetric intercellular metastasis, and cytotoxicity [6,28]. Exocytosis is also related to the translocation of NPs at the animal level. In nanomedicine, researchers design NPs as carriers to deliver therapeutic and imaging agents with the ultimate goal of achieving high-efficacy and high-sensitivity imaging [29]. To achieve this goal, NPs have to penetrate tissues to reach targets, and the retention of NPs in target cells is critical, as the discharge of NPs that have been engulfed ultimately affects the number of drugs at the site of action [28,30,31,32]. The longer the intracellular persistence time in target cells, the higher therapeutic potential of the NP-based drug carriers. It is therefore necessary to enhance the retention of NPs in cancerous cells and reduce their retention in healthy cells [33]. Yet the fate of these NP carriers (after releasing drugs) in cells is largely overlooked. There is a possibility that these NP carriers could be trapped in cells for a long time [22,28,34], and thereby induce cytotoxicity [16]. Therefore, understanding the process and mechanisms involved in the exocytosis of NPs is necessary and urgent [35].

Given the fact that exocytosis is an important yet less-studied area with few related review papers [3,6,7], we provide here a comprehensive review to summarize the experimental methods applied in the exocytosis study of NPs, current progresses on the exocytosis mechanisms/pathways and kinetics, and related influencing factors. We also discuss the problems existing in current studies and provide suggestions for research in the future to further enrich our knowledge of the exocytosis of NPs.

## 2. Experimental Methods

### 2.1. General Procedure and Consideration

**Figure 1:**
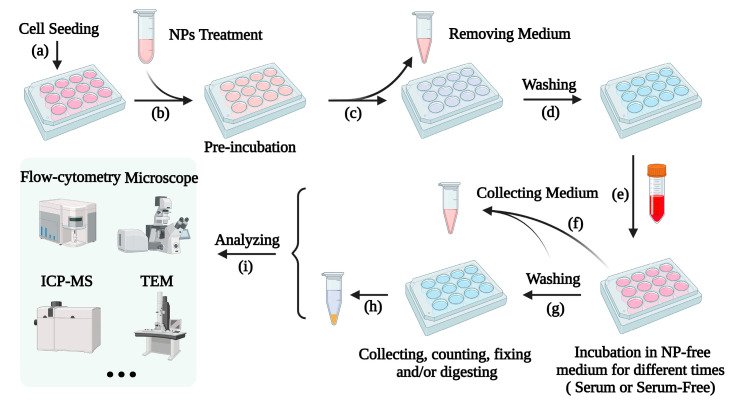
Schematic diagram of the common experimental process for studying the exocytosis of NPs. (a) Cells are seeded in plates/dishes; (b) Cells are incubated in medium containing NPs for different time periods; (c) Old medium is removed (collected if there is a need); (d) Cells are washed with buffer solutions like D-Hanks; (e) Fresh NP-free medium (containing serum or serum-free) is used to incubate cells; (f,g) At predetermined time intervals, the medium is collected if there is a need, and cells are washed again (buffer solution is collected and mixed with the collected medium if there is a need); (h) Cells are collected, counted, fixed, and/or digested; (i) Cells and/or the collected medium are measured by various methods.

Figure 1 illustrates the common procedure to study NP exocytosis. Generally, cells are firstly pretreated with NPs so that the cells can load enough NPs, then the extracellular NPs are washed away. Next, the NP-laden cells are incubated in fresh NP-free culture medium and the decrease in intracellular NPs and/or the increase in extracellular NPs over time are measured to reflect the efflux of NPs from the cells [6,36,37].

As the starting point for the study of exocytosis, the cellular content of NPs should be high enough to make sure the exocytosis process can be accurately detected. Most studies just pick a preincubation time, while few studies first measure the uptake course over time to determine the pre-uptake time; usually, the time point with the highest cellular content of NPs is chosen as the starting point for exocytosis investigation. In most cases, the NP-loading period is 2–6 h, and occasionally there is a much longer time, such as 72 h.

After NP loading and before exocytosis, extracellular NPs should be removed as much as possible by washing carefully with suitable buffer solutions or medium to make sure that residual NPs, such as the NPs attached to the surface of cells, do not impact the measurements of intracellular NP contents [38]. To eliminate such interference, some researchers detach the cells with trypsin and reseed the cells in new culture dishes [34,39]. This approach does greatly eliminate the interference of extracellular residual NPs, but it may affect the properties of cells and even damage the cell membrane, thereby altering the exocytosis of NPs. Another important issue to be considered is the cell density in plates/dishes, which is very critical for the long-term tracking of exocytosis [40].

After NP-laden cells are re-incubated in fresh NP-free culture medium, the NP excretion from the NP-laden cells can be recorded at different time intervals. Most studies monitor the exocytosis within 12 h, but a much longer time period has also been adopted. During this period, the culture medium is usually not changed. Replacing the medium (or performing in a fluid medium) will accelerate the exocytosis and promote the efflux of NPs from cells, leading to a more complete efflux because of the concentration gradient between NPs inside and outside the cells [12,14].

Changes in intracellular NP content during exocytosis can be detected directly or indirectly by various qualitative and quantitative analysis methods, including but not limited to fluorescence techniques, elemental analysis, and transmission electron microscopy (TEM), which will be fully detailed in the next section. It is important to note that the detected decrease in intracellular NPs contains the contribution of cell division, thus it does not equate to the net exocytosis of NPs [37,41]. So, sometimes extracellular NPs are collected to present the discharge of NPs from cells [11,42,43] which is not affected by cell division. However, the quantity of NPs released into a culture medium is generally pretty low and also affected by many other factors (such as adsorption by culture plates/dishes); completely collecting and measuring the NPs in culture media is very difficult [21,44]. Ideally, to obtain accurate exocytosis information, both the quantity of NPs inside and outside the cells should be analyzed during exocytosis [10,11,41], and the total intracellular NPs of all tested cells at the zero point should be equal to the sum of the NPs inside and outside the cells at any exocytosis point [11].

During the exocytosis period, cell division might be significant [37,41,45,46]. Cell division is the process of the redistribution of cell contents, including the NPs in the cells. After cell division, NPs in the mother cell are shared between the daughter cells [47,48]. Thus, cell division is also a major way to reduce the content of intracellular NPs (ignoring degradation) per cell [49]. The influence of cell division on the content of intracellular NPs depends on the proliferation speed of cells [50]. NPs in rapidly proliferating cells are diluted faster than in slowly proliferating cells, and the reduction in NPs in cells also influences the exocytosis of NPs because of the reduced concentration difference between intracellular and extracellular NPs.

To eliminate the effect of cell proliferation, besides completing the experiment before cells enter the division phase (such as completing preloading and exocytosis within 6 h), some researchers use inhibitors or culture cells in a serum-free medium to inhibit cell division [48,51], but these inhibitors may damage cells to some extent. Another approach is to calculate the changes in the total amount of NPs in all cultured cells by correcting with the cell number. Although the doubling time of cells could be used to estimate the cell number at a time point [41,52], cell growth is not always an ideal process, influenced by many factors, such as cell density. Therefore, our group choose to count cell numbers at detection time points, calculate the total amounts of NPs in all cultured cells, and compare them with the total amount of NPs in all cells at the starting point to obtain the absolute exocytosis of NPs in cells [36,37].

To reveal the exocytosis mechanisms/pathways, inhibitors/accelerators corresponding to different pathways can be used to illustrate the contribution of each pathway in the exocytosis of NPs. Table 1 lists the commonly used concentrations and functions of various exocytosis inhibitors/accelerators. It should be kept in mind that when these inhibitors/accelerators are used, their concentrations should be carefully chosen, as a much lower concentration cannot inhibit/accelerate the exocytosis process, while a higher concentration may cause damage to the cells, thereby altering the exocytosis of NPs. Therefore, it is a good practice to ensure that the inhibitors/accelerators do not affect the viability of the cells before use. Furthermore, intracellular NPs are usually excreted via more than one pathway, and some inhibitors may block more than one excretory pathway [37,53]. Therefore, it is suggested to apply inhibitors/promoters of different pathways, or even two inhibitors/promoters of the same pathway, to obtain accurate and complete information.

The exocytosis mechanisms/pathways of NPs can also be revealed by other approaches. One approach is by observing the colocalization of intracellular NPs and organelle labels/exocytosis pathway markers during the exocytosis process, which requires both NPs and markers being fluorescent/labeled. The common organelle labels include Lyso Tracker for lysosomes and Mito Tracker for mitochondria [37,44], and the common markers of trafficking vesicles include the early endosome marker Rab5, the late endosome marker Rab7, the transport marker from the late endosome to the Golgi (Rab24), the transport marker from the Golgi to the cell membrane (Rab3/Rab26), etc. [62,66,67]. Another approach is by measuring the specific molecules related to specific exocytosis routes. For instance, β-hexosaminidase is used as the marker of the lysosomal pathway [10,68] since it exists in lysosomes and can be expelled into a culture medium along with other substances. In addition, specific exocytosis mechanisms/pathways can also be identified by gene silencing/knocking down related key molecules [44,69].

### 2.2. Analytical Techniques

Both qualitative and quantitative techniques have been used to detect NPs to illustrate the exocytosis of NPs. Qualitative techniques are commonly used to track the transport and location of NPs in cells, while quantitative methods are used to measure the precise concentration variations in NPs during exocytosis. The combination of these methods may provide more comprehensive and accurate information about NPs in exocytosis.

The fluorescence method is the most common technique for both qualitative and quantitative estimations of NPs, including quantitative fluorescence spectrometry, quantitative flow cytometry, qualitative/semi-quantitative fluorescence microscopy, confocal laser scanning microscopy (CLSM), etc. All these methods rely on the fluorescence properties of NPs, which can be either the labeled dyes (such as fluorescein isothiocyanate (FITC) and rhodamine) on NPs or the intrinsic fluorescence of NPs (such as light-emitting materials–QDs [13,15] and upconversion luminescent materials [42]. The advantages of the labeling approach are its generality and flexibility, as a dye with appropriate excitation and emission properties can be chosen depending on the experimental need, although fluorescence labeling on the surface of NPs may alter the physiochemical properties of NPs, affecting the interaction between NPs and cells [70].

While fluorescence is usually specific and very sensitive, it is also susceptible to many external or internal factors that may lead to the loss of valid messages. For instance, the intracellular degradation of NPs results in the loss of fluorescence [10] or the detachment of labeled dyes, and the fluorescence signal may be quenched in different culture environments (such as acidic lysosomes), making the fluorescence unable to represent the NPs. Therefore, suitable controls and fluorescence stability tests of NPs in potentially exposed environments are required [42,48]. In addition, to obtain the absolute contents of NPs in cells, the fluorescence correction of NPs between in solution and in cells should be carefully set.

Fluorescence microscopy and CLSM are visual fluorescence techniques which can directly track NPs in cells. However, they are basically low-throughput localized techniques, thereby usually only a small number of cells can be monitored due to the limited investigation area. Flow cytometry is the most widely used fluorescence quantitative analysis technique to investigate the interaction between NPs and cells [10,12,58,71]. Flow cytometry also measures the fluorescence of individual cells, yet it is a high-throughput technique that provides the mean fluorescence intensity (MFI) per cell based on a large number of cells; thus, it can present the overall content of NPs in cells. However, flow cytometry cannot distinguish internal NPs from those attached to the membrane of cells.

Elemental analysis is an absolute quantitative method, which is an indispensable tool to determine the concentration of NPs inside and outside cells for those NPs that contain unique elements, such as Au and Ag. The flame/graphite furnace atomic absorption spectrometer, inductively coupled plasma mass spectrometer (ICP-MS) [12,15,58], and inductively coupled plasma optical emitted spectrometer (ICP-OES) [10,11] are the most common instruments for analyzing NPs without fluorescence but containing metal elements. To measure the content of NPs in cells, the cells need to be digested with acids or other reagents, and thus these techniques are destructive. Moreover, these techniques can only provide the average contents of metal elements in a large number of cells, which can be converted to the average content of NPs per cell. More importantly, these techniques are unable to distinguish the integrity of NPs (i.e., NPs and their dissolved ions), largely limiting their application in the detection of NPs. Recently, a single-particle ICP-MS and single-cell ICP-MS have been developed for the analysis of NPs in solutions and intracellular elements and NPs in single cells, respectively [72,73,74]. Although they can provide more detailed content information of NPs and NPs from individual cells, the sample preparation, measurement, and data analysis are complex, and a single-cell ICP-MS still cannot distinguish NPs and their dissolved ions. The quantitative analysis of NPs in cells remains a challenge in terms of accuracy.

TEM is a qualitative but high-resolution analysis technique to directly investigate intracellular NPs [14,34,75,76] and thus is usually used to be a supplementary method of fluorescence analysis and elemental analysis. TEM can clearly show the location of NPs and provide more direct evidence for the interaction between NPs and the cell membrane. For example, Chu et al. directly observed the slight decrease in 50 nm SiO_2_ NPs in NE083 cells in the first 1 h, followed by a dramatic decrease in the next 2 h, suggesting a time-dependent exocytosis process [9]. TEM is a microarea analysis technique, and TEM samples should not be thicker than 100 nm (the size of a cell is around 10 μm). Therefore, TEM can only observe NPs in some cell sections and cannot even observe all NPs in a complete cell [77], let alone NPs in a group of cells. In addition, TEM cannot discriminate between some NPs and other objects in the resulting image due to the poor contrast between the NPs and the background. To obtain quantitative data or general conclusions, carefully designed experiments and a large amount of statistically significant data are required. Direct TEM statistical results for the cellular uptake of NPs are available in some studies [78,79] but those for the exocytosis of NPs are still lacking.

Many techniques developed for other purposes can also be applied to investigate the exocytosis of NPs, such as western blot [45], high content analysis [80], optical imaging [81], and amperometry [82], although they are only used in few studies and have various limitations.

It should be emphasized that multiple analysis methods should generally be used together to obtain more reliable results and comprehensive conclusions on the exocytosis of NPs.

## 3. Exocytosis Mechanisms/Pathways of NPs

Exocytosis is the transport of nonessential molecules from the interior of a cell to the exterior to reduce the stress inside the cell, a process opposite to endocytosis. After NPs enter cells, they may reside and transport in various vesicles and organelles, including the endosome, lysosome, mitochondrion, ER/Golgi apparatus, nucleus, and cytoplasm [10,36,62,83,84]. Some NPs remain inside the cell for a long time without exocytosis [25,85], which is usually associated with cytotoxicity.

**Figure 2:**
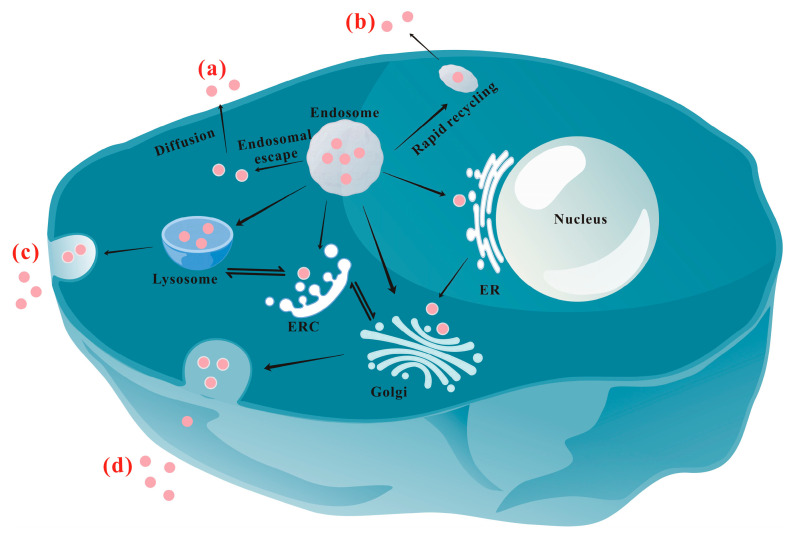
Schematic illustration of the main exocytosis mechanisms/pathways of NPs: (a) Diffusion; (b) Rapid recycling pathway; (c) Lysosomal pathway; (d) ER/Golgi apparatus pathway. ERC: endocytic recycling compartment; ER: endoplasmic reticulum.

Figure 2 schematically shows the main exocytosis mechanisms/pathways of NPs. At present, the recognized exocytosis pathways of NPs mainly include the following: (a) Diffusion: As an energy-independent pathway, diffusion allows NPs to cross the phospholipid membrane directly and passively [8,46,86,87]. Fundamentally, diffusion is driven by the concentration gradient of NPs between the inside and outside environment, and the coefficient of diffusion is inversely proportional to the diameter of the NPs. So, generally, only particles smaller than 50 nm have the possibility of entering and exiting the cell through diffusion [88]. NPs in the cytoplasm may be excreted via diffusion more slowly compared with vesicle-coated NPs [6,9,89], which are much easier to move within cells. The results are similar to those reported by Yang et al. [90], who found that more than 10 h was needed for the functionalized PS that first distributed in the cytoplasm and then moved to lysosomes to be eliminated by cells. (b) Rapid recycling pathway: Some NPs-containing early endosomes near the cell membrane may directly fuse with the membrane to rapidly release the NPs to the extracellular environment [44,91]. Researchers could compel NPs to exocytosis directly from the endosomes by inhibiting the transport of NPs to the perinuclear [53]. (c) Lysosomal pathway: This is the most important intracellular circulation pathway to release NPs to the extracellular environment [10,55]. Actually, both the rapid recycling and the lysosomal routes involve cell membrane fusion [92]. (d) ER/Golgi apparatus pathway: NPs delivered to the ER or Golgi apparatus are released by secreting vesicles that fuse with the cell membrane [93,94,95]. This is a relatively slow pathway [7,83,96,97]. (e) Other pathways: Special receptors on the surface of the cell membrane, such as P-glycoprotein and αvβ3 integrin, may interact with NPs to trigger exocytosis through rapid recycling of the receptors [71,98]. A survey of 70 available research papers on the exocytosis pathways of NPs reveals that the lysosomal pathway is the most important exocytosis mechanism of NPs (involving 46 papers). The second most important pathway is the ER/Golgi pathway, which is reported in 22 papers. Diffusion also holds a place, as reported in seven papers, while four papers reported different pathways from the above. Certainly, NPs may be excreted through more than one pathway, which was observed in 11 papers. In the following sections, we will introduce the lysosomal and ER/Golgi pathways in detail.

In addition, it is worth noting tubulin plays an important role in the exocytosis of NPs. In fact, the microtubule acts as a kind of “courier” that is involved in the whole process of exocytosis [42,58,99].

### 3.1. Lysosomal Pathway

The lysosome is the terminal degradative compartment of the endocytic pathway. After internalization, most NPs will be eventually trapped in lysosomes [9,10,15]. As shown in Figure 2, most of the NP-containing endosomes will mature into lysosomes, where some NPs might be degraded. If not degraded, cargoes in the lysosomal system are expected to be recycled back to the cell surface [45]. Stable NPs in lysosomes would either enter the Golgi apparatus via the endocytic recycling compartment (ERC) for excretion or undergo lysosomal exocytosis directly [100]. It has also been reported that inhibiting the autophagy could prevent the accumulation of polystyrene (PS) NPs in lysosomes and thereby egress in live alveolar epithelial cells [55], indicating NPs in autophagic vacuoles could be excreted via the lysosomal pathway after autophagic vacuoles fuse with lysosomes [66,101].

**Figure 3:**
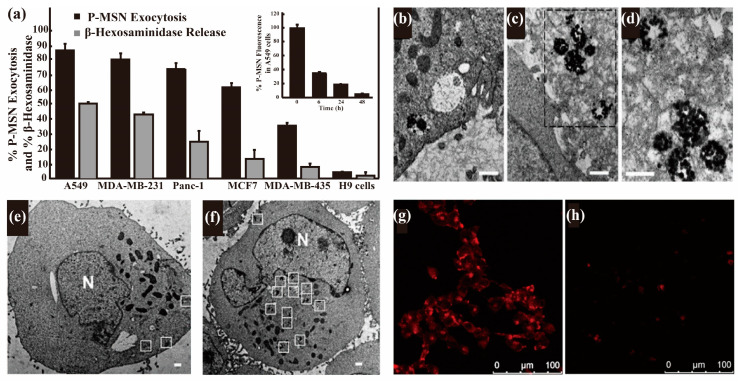
The exocytosis of different SiO_2_ NPs from different cells. (**a**) The exocytosis of P-MSNs over time in A549 cells after 2 h preuptake incubation, and the exocytosis of P-MSNs and the secretion of β-hexosaminidase in different cell lines at 6 h after 2 h preuptake incubation (reproduced from [10] with permission of John Wiley & Sons); (**b**) An NP-containing vesicle inside the cell close to the cell membrane; (**c**) NP-containing vesicles outside the cell; (**d**) Magnification of the rectangle area in (**c**), showing that these vesicles are membrane bounded; (**e**) and (**g**), TEM image and CLSM image (**g**) of a cell treated with NPs for 48 h in a serum-free medium; (**f**) and (**h**), TEM image (**f**) and CLSM image (**h**) taken from the same sample after an additional 1 h incubation in a fresh NP- and serum-free medium. The rectangles indicate the location of NPs; the scale bar is 500 nm; N stands for nucleus (reproduced from [9] with permission of The Royal Society of Chemistry).

The vesicle-packed NPs are excreted from the lysosome when the vesicle approaches and fuses with the cell membrane [10]. For example, Yanes et al. found that the efflux of mesoporous SiO_2_ NPs (MSNs) in several types of cells was mainly through lysosomal exocytosis [10]. Phosphonate-modified MSNs (P-MSNs) in the cells dramatically decreased to less than 40% of the control after 6 h in the P-MSN-free medium, and almost all intracellular MSNs were excreted after 48 h (Figure 3a). The exocytosis in different cells could be inhibited by nocodazole (Table 1, inhibit the transport of the lysosome to the periphery and fusion with the plasma membrane) and the excretion of P-MSNs positively correlated to that of the lysosome secretion marker β-hexosaminidase (Figure 3a), indicating it is a lysosomal exocytosis process. Similarly, Chu et al. systematically studied the excretion of 50 nm SiO_2_ NPs by preincubating cells with NPs for 48 h and then incubating them in an NP-free medium for 1 h [9]. TEM investigation indicated that, after 48 h incubation, the exocytosis of NPs occurred along with endocytosis (Figure 3b–d), and most NPs accumulated in lysosomes (Figure 3e). When the NP-laden cells were cultured for 1 h in an NP- and serum-free medium, the number of NPs in lysosomes decreased dramatically (Figure 3f), suggesting the excretion of NPs through the lysosomal pathway. The TEM result was confirmed by the decrease in the fluorescence intensity of NPs in cells measured using CLSM (Figure 3g–h). The exocytosis of functionalized MSNs in 4T1 cells was also mediated by the lysosomal pathway rather than the Golgi apparatus pathway because Brefeldin A (Table 1, causes the collapse of the Golgi apparatus) did not inhibit the exocytosis of MSNs [102]. Liu et al. reported that the exocytosis of 50 nm PS and 500 nm PS were decreased to 33% and 40% of the control group, respectively, by the inhibitor Bafilomycin A1, while the exocytosis was promoted to 125% (50 nm PS) and 148% (500 nm PS) by the accelerator ionomycin [103], confirming the lysosomal exocytosis of these PSs. Similarly, chemical inhibition studies have confirmed that the lysosomal pathway is the main exocytosis pathway for polyethylene glycol (PEG)-phospholipid-coated upconversion NPs in HeLa cells [42] and polyamidoamine (PAMAM-NH_2_) NPs in MCF-7/ADR cells [56].

It should be noted that the decrease in NPs in lysosomes cannot be directly attributed to the exocytosis of NPs via the lysosomal pathway. NPs in lysosomes may be delivered to other organelles, such as the Golgi apparatus (Figure 2) [37,44,83]. In addition, NPs in lysosomes may undergo degradation (such as the detachment of surface modification groups) and then can hardly be involved in exocytosis or intracellular trafficking, resulting in a longer intracellular retention [32,104].

### 3.2. ER/Golgi Pathway

**Figure 4:**
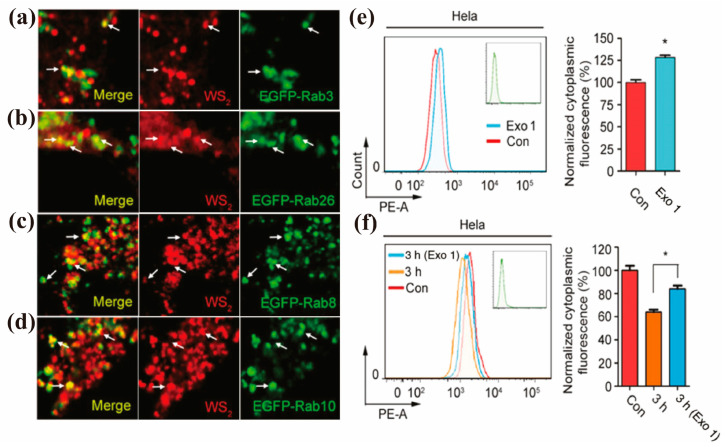
Exocytosis of WS_2_ NSs in HeLa cells via the ER/Golgi pathway. (**a**–**d**) HeLa cells were transfected with EGFP-Rab3 plasmid (**a**), EGFP-Rab26 plasmid (**b**), EGFP-Rab8 plasmid (**c**), and EGFP-Rab10 plasmid (**d**), respectively, and then the cells were incubated with RB-labeled WS_2_ NSs for 2 h. The white arrows points the co-localization of Rab-labelled vesicles with WS_2_ NSs. Scale bars: 10 μm. (**e**) HeLa cells were pretreated with Exo1 for 2 h, followed by 3 h of incubation with rhodamine-labeled WS_2_ NSs. (**f**) HeLa cells were first incubated with rhodamine-labeled WS_2_ NSs for 3 h, followed by 2 h of Exo1 treatment. Then, the medium was removed, and the cells were kept in fresh medium for 3 h (reproduced from [83] with permission of De Gruyter). The data are presented as mean ± SEM in these studies (*, *p* < 0.05).

In addition to lysosomal exocytosis, the secretory pathway containing the Golgi apparatus (and ER) is another important exocytosis route [32,105]. In this pathway, cells eject ingested contents through certain specific secretory vesicles, which is also believed to be a safe route for drug delivery that escapes the degradation of lysosomes [106]. NPs transported from the ER or other different places such as lysosomes first arrive at the Golgi apparatus and then are transported to the cell membrane. In the literature, some studies may only report that NPs are excreted via the Golgi apparatus, without analyzing whether they pass through the ER. For example, by using vesicle marker GTPases to investigate the colocalization of WS_2_ nanosheets (NSs) with different vesicles, Kong et al. found that WS_2_ NSs in early and late endosomes (Rab22- and Rab24-labeled, respectively) might be delivered to the Golgi, and then the WS_2_ NSs could be excreted into the extracellular space with the help of classic (Rab26- and Rab3-labeled) and GLUT4 (Rab10- and Rab8-labeled) translocated vesicles (Figure 4a–d) [83]. The excretion mechanism was confirmed by inhibiting the exocytosis using the exocytosis inhibitor Exo1 (Figure 4e–f), which induces the collapse of the Golgi apparatus (Table 1). Similarly, Ding et al. reported that Exo1 inhibited the excretion of intracellular fluorescent MSNs, and the fluorescent MSNs merged with Rab3 and Rab26 (marking the transfer from the Golgi to the cell membrane) marked secretory vesicles well shown by CLSM, indicating the involvement of the Golgi exocytosis pathway in their efflux [62].

Whether NPs pass through the ER to the Golgi and then are expelled can be identified by using specific chemical inhibitors or trafficking vesicle markers (such as Rab1, Rab2, and Rab43) [56,66]. For example, Liu et al. found that the discharge of intracellular CdSe/ZnS QDs functionalized with 2-mercaptoethylamine (MEA-QDs) could be dramatically suppressed when Brefeldin A and Monensin were used to block the ER and Golgi pathways, respectively, indicating that both the ER and Golgi apparatus are crucial regulatory stations in the exocytosis of MEA-QDs [37]. Xing et al. observed that the inhibitor Brefeldin A could block the exocytosis process of L-cysteine (Cys) and cell-penetrating peptide octa-arginine (R8)-modified insulin-loaded NPs, suggesting that the NPs were excreted via the pathway from the ER/Golgi apparatus to the cell surface [99]. Further studies indicated that Cys modification was critical for the exocytosis through the Golgi secretory pathway due to the thiol group of Cys binding to the sulfhydryl receptor site in the Golgi apparatus.

The golgi apparatus is an important regulator which participates in the ER/Golgi apparatus pathway and Golgi apparatus/plasma membrane pathway. During the process of exocytosis, the ER and Golgi apparatus secrete some NP-containing vesicles, while the vesicle-SNARE (v-SNARE) protein interacts with the target-SNARE (t-SNARE) protein in the plasma membrane, followed by further membrane fusion [107,108]. As the proteins widely spread in the plasma membrane–Golgi–ER recycling pathway, SNARE proteins are responsible for membrane fusion [32]. Lysosomal exocytosis also involves a similar process and corresponding SNARE proteins [109].

### 3.3. The Auxiliary Effect of the Microtubule

Except for passive diffusion, the microtubule is involved in all the other exocytosis pathways of NPs. During exocytosis, NPs in endosomes, lysosomes, the ER/Golgi, or other compartments move out of the cell with the help of microtubules [13,42,62]. In fact, ERC is a microtubular organelle distributed in the cytoplasm, which is an important chamber that takes part in the formation, fusion, and release of vesicles. NPs transported to the ERC can be recycled back to the plasma membrane via recycling vesicles and/or be directed to lysosomes, the ER/Golgi apparatus, and other organelles.

In general, NPs encapsulated in vesicle systems are delivered from the cell periphery to the perinuclear region by dyneins along microtubules, while their delivery from the perinuclear region to the cell periphery is operated by kinesins along microtubules [110,111]. In the excretion process, NPs accumulated in microtubules near the nucleus gradually disperse from the accumulation site and then travel along microtubules to nearby cell membranes [112], where they are expelled [42]. For example, Cao et al. reported that most of Si-NPs located in early endosomes were transported to lysosomes and the ER/Golgi with the aid of microtubules [67]. Liu et al. also observed that the exocytosis of carbon dots (CDs) in five types of cells was microtubule-dependent [37], since Nocodazole significantly inhibited the efflux of CDs from the cells. Malik et al. studied the trafficking and exocytosis of PNA (peptide nucleic acids, 180 nm) in HeLa cells using CLSM and flow cytometry and found that the interruption of the recycling pathway of PNAs via inhibiting either Rab11a or Rab27b directed the intracellular trafficking toward the lysosomal stage, while Rab11a and Rab27b were associated with microtubule organelle ERC [44].

The pathways chosen by a cell to eliminate NPs are influenced by a variety of factors, including the physiochemical properties of NPs, cell type, extracellular conditions, etc., which will be discussed later in this review. Currently, there is no widely recognized conclusion about which parameters regulate the intracellular NPs to leave cells via specific pathways. Basically, the uptake pathways of NPs decide their exocytosis in a specific cell line. For example, NPs entering cells via caveolin-mediated endocytosis mainly enter the ER, and then most of them leave the cells via the ER/Golgi apparatus. However, some of them may also leave the ER and enter lysosomes, following exocytosis via lysosomal exocytosis. Exocytosis is complex and the related mechanisms need further in-depth research.

It is worth noting that exocytosis experiments are generally performed under nontoxic conditions to eliminate the influence induced by cell damage [37]. The cytotoxicity of NPs has potential effects on exocytosis, for example, we can reasonably speculate that damage to the cell membrane may accelerate the exocytosis of NPs. Gupta et al. [113] reported that nanosized extracellular vesicles were excreted from normal cells through Rab protein-dependent lysosomal exocytosis, but those vesicles were excreted from necroptotic cells through the mixed-lineage kinase-domain-like (MLKL)-mediated calcium influx induced lysosomal exocytosis (Rab protein-independent), indicating the status of cells affects the exocytosis process. However, as cytotoxicity is complex and difficult to be precisely regulated, exocytosis studies are performed generally on cells without an obvious viability loss, though it is interesting to investigate the effect of cytotoxicity on the exocytosis of NPs.

## 4. Kinetics of the Exocytosis of NPs

Generally, the endocytosis and exocytosis process of NPs are not independent processes but always occur simultaneously [9,11], and their dominance in nano–bio interactions transforms as the intracellular concentration of NPs changes. At the beginning of exposure to NPs, endocytosis dominates in cells. As the content of intracellular NPs increases over time, the exocytosis of NPs appears [37] and increases gradually to reach a balance with the endocytosis of NPs until the intracellular NP content reaches saturation. At this time, the endocytosis and exocytosis jointly maintain the content of intracellular NPs [36,114,115,116], although Ho et al. reported there was limited NP exocytosis during endocytosis [76]. Subsequently, the content of intracellular NPs may drop, probably because the exocytosis has an advantage at that moment [10,36,37,115,116]. Another plausible explanation is the lag time between the endocytosis and exocytosis processes which can make an overshot of the net content of NPs and then return to the balance point.

Similarly, at the beginning of the re-incubation of NP-laden cells in an NP-free medium, the big concentration difference between NPs inside and outside the cell makes the excretion process dominant first, then the reuptake of NPs emerges and gradually reaches the dynamic equilibrium between NPs inside and outside of the cell [117,118], though the NP re-endocytosis is limited during exocytosis [76]. Interestingly, the released NPs from one cell can be internalized again by another cell [87], known as transport exocytosis [57].

Basically, the exocytosis of NPs is a highly dynamic complicated process involving multiple pathways, and the exocytosis is dependent on the concentration difference of intracellular and extracellular NPs [36,37]. Generally, it is more convenient to investigate the exocytosis of NPs with fully NP-laden cells re-incubated in NP-free media. In this case, the discharge rate in the initial stage is higher than that in the subsequent stages [36,37,42,119]. As time goes on, the discharge rate slows down until the maximum reduction is reached. The time needed to reach the maximum exocytosis in most reports is 4–6 h, but it is greatly influenced by the properties of NPs, cell type, and culture conditions [41]. Chithrani et al. reported that the 14 nm transferrin-coated Au NPs had a faster exocytosis rate (removal half-life was 0.33–0.41 h) in HeLa, STO, and SNB19 cells than the 50 nm NPs (removal half-life was 0.41–0.58 h) and 74 nm NPs (removal half-life was 0.58–0.75 h) since smaller NPs had less receptor–ligand interactions [75]. A similar result was reported by Bonamy et al. [120] and the 14 nm transferrin-coated Au NPs also had a higher exocytosis amount in these cells compared to the bigger ones, which is consistent with studies in other cell lines [52]. Moreover, the rod-shaped NPs exhibited a higher exocytosis amount (around 80% of the control) in HeLa and SNB19 cells than the spherical NPs (around 20–40% of the control) [75]. However, Wang et al. found that rod-shaped serum-protein-coated Au NPs were hardly excreted by A549 and 16 HBE cells even in an NP-free culture medium for 72 h, while about 60% of the intracellular NPs was excluded in the MSC cells [28].

It is important to determine how the NP content per cell changes during the exocytosis period [121]. A key point here is that the change in intracellular NP content may be caused by not only the efflux of NPs but also by cell division, which can be distinguished by measuring the extracellular NP content or by calculating changes in the total amount of intracellular NPs in all cells [36,37,52]. Long-term toxic effects of NPs depend on the residuals of NPs after accomplishing exocytosis or whether NPs can be degraded [122].

Besides the NP content changes over time, the organelle distribution of NPs in cells also changes over time, which has a close relationship with exocytosis. However, at present, data on the translocation kinetics of NPs between organelles are still scarce because the exocytosis process of NPs involves multiple pathways that intersect with each other, and it is difficult to measure exactly how many NPs are transported from one specific organelle to another organelle in a specific time by imaging the colocalization of NPs and organelles. For example, we found that some QDs moved out of lysosomes at 1 h after exocytosis, but we did not know which organelles these QDs entered (we did not label all kinds of organelles) and could not measure the exact amount in organelles (at most, it is a semi-quantitative analysis) [36]. More attention should be paid to this issue in future studies.

## 5. Influencing Factors

Many factors influence the exocytosis of NPs, including the mechanisms/pathways and the amount or portion of NPs that can be finally excreted. Table 2 is a summary of the studies on the efflux of typical NPs. The data show that the exocytosis of NPs is strongly influenced by various factors, including the physicochemical properties of NPs (size, shape, and surface groups), cell type, and cell culture conditions [42].

### 5.1. Intracellular Content and Location of NPs at the Initial Stage of Exocytosis

**Figure 5:**
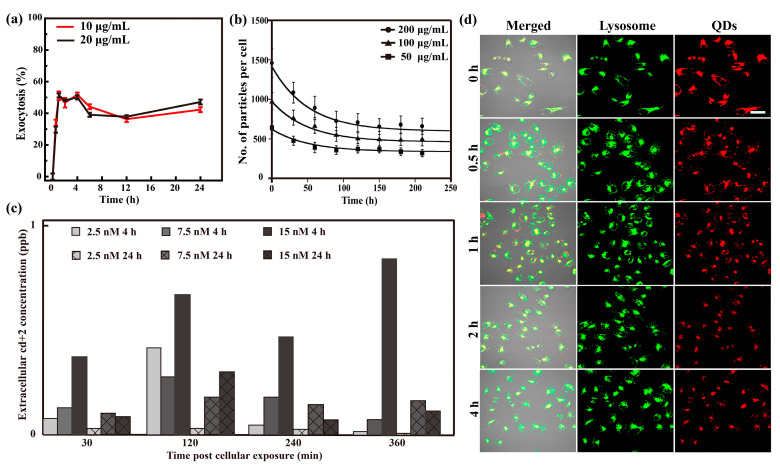
Effect of initial intracellular content and location of NPs on the exocytosis of NPs. (**a**) Exocytosis of CDs-PEI in HeLa cells over time after the cells were pretreated with 10 μg/mL and 20 μg/mL of CDs-PEI for 4 h (reproduced from [37] with permission of the American Chemical Society); (**b**) Exocytosis of Si QDs in HUVEC cells over time after the cells were preincubated with Si QDs of 200 mg/mL (circles), 100 mg/mL (triangles), and 50 mg/mL (squares) for 4 h. Solid lines represent the results of the model proposed (reproduced from [14] with permission of Elsevier); (**c**) The exocytosis of NH_2_-QDs (the Cd content culture medium) at different time points after the cells were preincubated with NH_2_-QDs of different concentrations for 4 h (solid filled bars) and 24 h (dotted bars) [25]; (**d**) CLSM images of MEA-QDs in lysosomes during exocytosis after HeLa cells were pretreated with 25 μg/mL MEA-QDs for 6 h. The scale represents 40 μm (reproduced from [36] with permission of Elsevier).

Based on limited data, there is no unified conclusion on whether the exocytosis process depends on the initial concentration of intracellular NPs [34,81]. In some reports, the initial concentration of intracellular NPs does not affect the exocytosis rate and relative exocytosis amount [14,37]. Liu et al. investigated the exocytosis of CDs in five cell lines after the cells were pretreated with 10 μg/mL and 20 μg/mL CDs-PEI (Figure 5a) [37]. The results showed that, although the initial intracellular content of CDs doubled when the concentration of CDs increased from 10 μg/mL to 20 μg/mL, the exocytosis rates and maximum exocytosis percentages in each cell line were almost the same under the two concentrations. This is similar to what Ohta et al. reported [14], that is, although the residual contents of Si QDs in the cells were different, around 40% of internalized Si QDs were excreted after the cells were preincubated with Si QDs of different concentrations (Figure 5b). Ho et al. also reported that a higher cellular uptake of NPs does not guarantee a higher exocytosis percentage [76]. However, different results have been reported by other researchers [25,34,119]. Manshian et al. concluded that both the preincubation dose and time affected the subsequent exocytosis of QDs (Figure 5c) [25]. Panyam and Labhasetwar found that it was the preincubation dose but not the preincubation time that affected the exocytosis percentage of poly(d,l-lactide-co-glycolide) (PLGA) in the vascular smooth muscle cell line YSMC [34]. Hu et al. reported that the preincubation time is important [119]. When it was extended from 12 h to 24 h, the exocytosis percentages decreased from 63% to 50% and from 38% to 10% for 60 nm and 600 nm SiO_2_ NPs, respectively.

The fate of NPs inside the cell is largely controlled by their localization [8,55], which affects the way that the cell eliminates them through exocytosis or degradation [42,44]. Liu et al. found that the location of CDs obtained from the carbonization of citric acid with polyethylenimine (PEI) oligomers (CDs-PEI) at the beginning of exocytosis was not correlated with their exocytosis pathway [37], suggesting that changes in the concentration of NPs at one organelle may be due to the transport of NPs to other sites rather than exocytosis. In the exocytosis study of MEA-QDs, Liu et al. also observed that the QDs initially accumulated in lysosomes decreased over time (Figure 5d). Some of the decrease was due to efflux, while others only left lysosomes but were still in the cells (Figure 5d), suggesting not all QDs in lysosomes would directly leave cells via the lysosomal pathway [36]. A similar phenomenon was observed by He et al. [17,20]. In another study, Strobel et al. found CeO_2_ NPs partly in the cytosol and rarely located in lysosomes, but the exocytosis was blocked by the inhibitor MβCD (Table 1, inhibits the plasma membrane cholesterol and Golgi to cell surface transport) [58]. Moreover, Wang et al. reported that most Au NRs in MSC cells were resident in lysosomes and were finally cleared, while Au NRs in A549 cells were difficult to be excluded due to their translocation from endosomes/lysosomes to mitochondria [28]. These results demonstrate NPs can be transported in different subcellular structures, and the initial location of NPs before exocytosis may not directly point to their exocytosis pathway, which may be influenced by NPs, cell type, or other factors.

With the help of fluorescent markers and some inhibitors, we can qualitatively analyze the transport of NPs in cells, but it is difficult to accurately measure the amount of variation in the heterozygous organelles. More attention should be paid to revealing the extent to which the organelles involved in the efflux contribute to the overall exocytosis process.

In most exocytosis studies, researchers just pick a preincubation time and a NP concentration to let cells engulf NPs and then perform the exocytosis in the NP-free culture medium (Table 2), i.e., the intracellular content of NPs for exocytosis may not be the highest content of NPs in cells, and the locations of NPs in cells may not the destination of NPs. Then, the exocytosis of NPs with different preincubation doses and times may be much different. More attention should be paid to investigate the influence of intracellular content and the location of NPs on their exocytosis.

### 5.2. NP-Related Factors

Relatively speaking, there is a wealth of data on how the physiochemical properties of NPs, such as size, shape, and surface modification, affect their exocytosis progression. But apart from the size effect, researchers have not reached a consensus on this issue. The reasons are not only that the properties of NPs have many aspects, which are interrelated (for instance, the change in shape interrelates to the size and mass of NPs), but also due to the influence of factors other than NPs (such as cell type). The key factors reported include the size, shape, and surface modification of NPs. Generally, it is assumed that the size, shape, and surface modification of NPs affect the quantity and distributions of NPs in cells, thereby leading to different exocytosis behaviors.

It has to be pointed out that once entering the biosystems, NPs will quickly be coated with proteins to form a shell of protein corona, and it is the NPs and the corona as a whole that determine the cellular behavior and fate of NPs [125]. As the formation of the protein corona is mainly due to a nonspecific interaction, and it is a highly dynamic, ever-changing process [125], it is very difficult to distinguish the real effects of certain factors of NPs per se. For the endocytosis study, it is possible to simplify the problem by incubating cells in an NP-containing serum-free medium, so as to prevent the formation of the protein corona on NPs before them entering cells [126]. However, for the exocytosis study, it is inevitable for the NPs to form the corona with proteins inside the cells. Since it is almost impossible to characterize the protein corona of NPs inside cells without destroying the cells, most studies on exocytosis did not characterize the corona. In the following discussions, we should be aware of the fact that the protein corona is almost always present, and we should be cautious of their conclusions.

In addition, the stability of NPs (the dissolution of NPs per se and the detachment of surface modification groups) in cells should not be neglected. Wang et al. [127] investigated the intracellular fate of Au@CuO NPs in different cells. They found that the dissolved Cu ions induced ROS overproduction and then were rapidly eliminated from the cells via a GSH-mediated pathway and lysosome exocytosis. However, the gold cores retained in cells for a long time. Besides interfering with NP detection [10], the dissolution of NPs not only may change the size and/or number of NPs but also may induce cytotoxicity, which alters the exocytosis of NPs [113]. Chen et al. reported that the detachment of PEG from PEG-functionalized NPs trapped in lysosomes resulted in an enhanced intracellular retention of NPs [32], indicating that surface group detachment may also influence the exocytosis of NPs and the detection of exocytosis [122].

#### 5.2.1. Size

**Figure 6:**
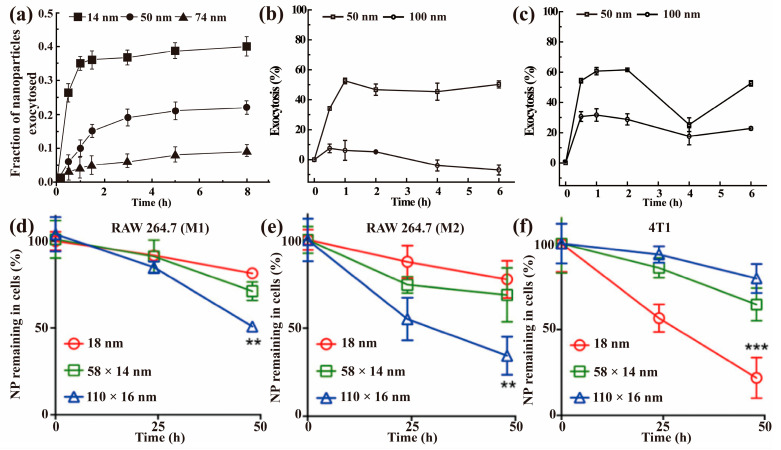
Effect of size and shape on the exocytosis of NPs. (**a**) Exocytosis of Au NPs of different sizes in HeLa cells (reproduced from [75] with permission of the American Chemical Society); (**b**,**c**) Exocytosis of 50 nm PS NPs and 100 nm PS NPs in cell line A549 (**b**) and BEAS-2B (**c**) [124]; (**d**,**f**) Exocytosis of Au NPs of different shapes in M1-polarized Raw264.7 (**d**), M2-polarized Raw264.7 (**e**), and 4T1 (**f**) cells after pretreatment with Au NPs for 6 h (reproduced from [68] with permission of the American Chemical Society). Data represent the mean ± SD (n = 3, ** *p* < 0.01, and *** *p* < 0.001).

Studies have demonstrated that the size of NPs can significantly affect the uptake rate and quantity of NPs by cells, with NPs of 50 nm being the most easily internalized by cells [5,126,128]. Not surprisingly, the exocytosis of NPs also is size-dependent but with smaller NPs being more easily excreted by cells [8,15,22,75]. Chithrani et al. reported that the excretion rate of 14 nm transferrin-coated Au NPs was twice that of 50 nm Au NPs, and the exocytosis fraction of 14 nm Au NPs was five-fold higher than that of 74 nm size NPs (Figure 6a) [75]. A more plausible explanation was that small particles had less receptor–ligand interactions compared to big ones, leading to an easier release and faster removal [53]. A similar result was obtained by Liu et al. [124], who found that 50 nm PS NPs were easier to be excreted from both A549 and BEAS-2B cells than 100 nm PS NPs, and 100 nm PS NPs almost did not leave A549 cells (Figure 6b–c). Serda et al. measured the release of superparamagnetic iron oxide NPs (SPIONs) from J774 cells and found that 15 nm NPs were easier to be excreted than 30 nm NPs [8], indicating a greater release of smaller SPIONs. Hu et al. studied the exocytosis of SiO_2_ NPs of different sizes in Hep-G2 cells within 12 h after preloading for 12 h [119]. They found that the final exocytosis percentages for 60, 180, 370, and 600 nm NPs were 63%, 67%, 58%, and 38% of the control, respectively, indicating that smaller SiO_2_ NPs tended to undergo exocytosis at a faster rate and in a higher amount compared to bigger ones. Such a size effect was also demonstrated on amino-capping QDs by Peng et al. [15]. They concluded that the elimination rate of the QDs decreased as the QDs size increased by comparing the three QDs (17.1 nm NH_2_-525, 18.9 nm NH_2_-585, and 22.8 nm NH_2_-625).

Ohta et al. developed a kinetic modeling to analyze the removal of Si QDs in which the dissociation of Si QDs from the receptors in acidic endosomes was used as a key indicator of the QD elimination [14]. They demonstrated that the change in this dissociation constant depended on particle size, which decided how many particles could be released out of the cell after cultured in a fresh medium. The size of NPs can affect the exocytosis of NPs by affecting the subcellular distribution of NPs after internalization by cells [6,28,124]. Liu et al. reported that 50 nm PS mainly appeared in lysosomes, while 100 nm PS accumulated in both lysosomes and mitochondria before exocytosis [124]. This may be one of the reasons why 100 nm PS NPs were harder to be excreted than 50 nm PS NPs. In addition, the exocytosis of NPs is energy dependent [9,11], and thus big particles require more transport proteins and a greater energy supply to leave the cell.

#### 5.2.2. Shape

The shape effect on the endocytosis of NPs has been reported in many studies [75,129,130]. However, only few studies focus on the shape influence on the exocytosis of NPs. Zhuang et al. found nanorods (100 × 400 nm) had a lower efflux rate and quantity in Caco-2 cells compared with nanospheres (180 nm) [131]. Differently, Oh et al. studied the exocytosis of three kinds of PEG-coated Au NPs (18 nm, 14 × 58 nm, and 16 × 110 nm) in M1/M2-polarized Raw264.7 and 4T1 cells within 48 h after pre-treatment with Au NPs for 6 h. It was found that spherical 18 nm Au NPs were excreted more efficiently than other two rod Au NPs in 4T1 cells, but they displayed opposite trends in M1/M2-polarized Raw264.7 cells (Figure 6d–f) [68]. Interestingly, These NPs exited all kinds of cells mainly via lysosomal exocytosis, as shown by the similar exocytosis patterns of NPs and β-hexosaminidase [68]. Similarly, Chithrani et al. found that the exocytosis rates of rod-shaped transferrin-coated Au NPs (20 × 30 nm, 14 × 50 nm, and 7 × 42 nm) was much higher than those of spherical-shaped ones (14 nm, 50 nm, and 74 nm) in HeLa and SNB 19 cells, but rod- and spherical-shaped NPs had the same release rate in STO cells [75]. The inconsistent results indicate cell type also play a role in shape effect.

Similar to size, it is assumed that shape can also affect the distribution of NPs in cells, thereby leading to different exocytosis behaviors. However, currently, there are no direct data to support it. Although Oh et al. reported that Au NPs with different shapes had different excretion behaviors in different cells, they did not investigate the initial location of these NPs before exocytosis [68]. Huang et al. investigated the effect of the shape of MSNs on cellular uptake [132]. The CLSM images showed that the rod particles (120 × 240 nm, and 110 × 450 nm) were closer to the nuclear region of A375 cells than the spherical particles (100 nm). This may be one reason why spherical particles were easier to be excreted, as NPs near the membrane are rapidly excreted [55]. More attention should be paid to this issue. In addition, nonspherical NPs are more easily clipping vesicle membranes and escaping to the cytoplasm, thereby reducing the cellular excretion rate of NPs [133]. Although the transport and fate of NPs retained in cytosol have not been well understood, the entry of NPs into the cytoplasm through endosomal escape or other means is crucial for prolonging the retention time of NPs in cells [134].

#### 5.2.3. Surface Modification

One advantage of NPs is easy surface modification by peptides, proteins or other organic molecules, which is used to improve the dispersity and stability of NPs in medium and other desired properties, such as, to enhance the interaction of NPs with specific cells. Surface modification mainly changes surface group and/or surface charge of NPs. Although the research is not yet in-depth, surface modification does affect the exocytosis of NPs [11,116].

**Figure 7:**
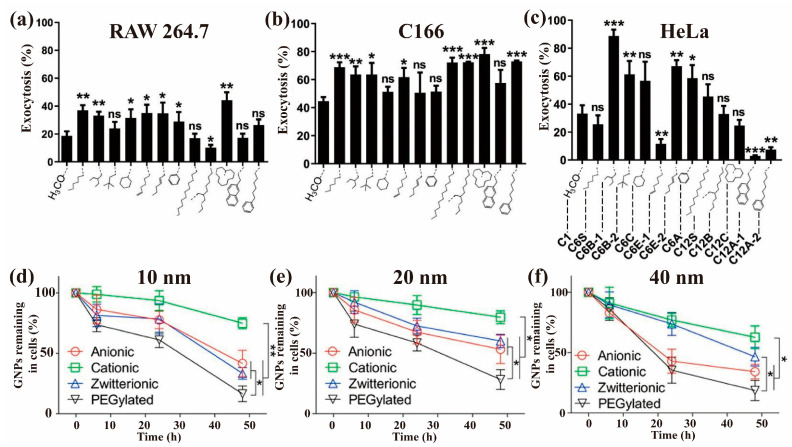
Effect of surface modification on the exocytosis of NPs. (**a**–**c**) Exocytosis of Au@PEG-X NPs in cell line Raw264.7 (**a**), C166 (**b**), and HeLa (**c**) within 24 h after the cells were pretreated with Au@PEG-X NPs for 6 h. X represents the hydrocarbyl groups attached to the end of PEG (reproduced from [116] with permission of the American Chemical Society); (**d**,**f**) Exocytosis of serum-coated Au NPs with sizes of 10 nm (**d**), 20 nm (**e**), and 40 nm (**f**) in macrophages after preincubation with the Au NPs for 6 h (reproduced from [123] with permission of the American Chemical Society). Data represent mean ± SD (n = 3, * *p* < 0.05, ** *p* < 0.01 and *** *p* < 0.001).

Ho et al. studied the exocytosis of 12 hydrophilic Au@PEG NPs (25 nm Au NP, and PEG with different hydrocarbyl groups as end groups) in Raw264.7, C166, and HeLa cells (Figure 7a–c) [116]. The results showed that surface groups markedly affected the exocytosis of NPs, especially in HeLa cells. In HeLa cells, the highest exocytosis percentage was over 90% for C6B-1, while the lowest was around 5% for C12A-1. In the study of the release of 100 nm MSNs from A549 cells [10], it was observed that 84% of P-MSNs was excreted after 6 h, while the excretion percentages of folate modified MSNs and PEI modified MSNs were 66% and 49%, respectively.

Besides types of surface groups, the components and density of the surface groups of NPs also influences the exocytosis behaviors of NPs. Xing et al. found that the exocytosis pathways of NPs modified with R8 (octa-arginine) and Cys (L-cysteine) in Caco-2 cells varied with the proportion of R8 and Cys on NPs [99]. Compared with 50% R8/50% Cys NPs and 75% R8/25% Cys NPs, 25% R8/75% Cys NPs could more effectively avoid lysosomal exocytosis and prone to higher Golgi exocytosis. Dalal et al. reported that the density of TAT peptide modified on QDs (30–35 nm) controlled the subcellular location and exocytosis mechanism of QDs [135]. The QDs with peptide densities of 40 and 75 were trapped in vesicles and expelled within 12 h, while lower peptide density of 10 and 20 offered the QDs efficient trafficking toward perinuclear region and Golgi apparatus, which made NPs retain in the cell for a longer time.

Surface modification usually changes the charge of NPs, which may lead to different exocytosis processes. Oh et al. found positively-charged Au NPs were harder to be excreted than negatively-charged ones in macrophages (Figure 7d–f), regardless of particle size, because positively charged Au NPs agglomerated inside the cells, which slowed down the transport of Au NPs and thus decreased exocytosis [134]. In contrast, Zhang et al. reported an exocytosis ability in decreasing order of positively-charged PAMAM-NH_2_ > neutral PAMAM-COOH > negatively-charged PAMAM-OH in MCF-7/ADR cells [56]. The exocytosis ability in decreasing order was PAMAM-NH_2_ > PAMAM-COOH > PAMAM-OH. Manshian et al. found COOH-QDs resulted in a much higher cellular content than NH_2_-QDs after incubation with HFF-1 cells for 4 h, but the extracellular contents of NH_2_-QDs was much higher than that of COOH-QDs after subsequent exocytosis for 4 h [25], indicating NH_2_-QDs were much more easily to be released from the cells than COOH-QDs. In addition, the exocytosis of COOH-QDs displayed a dose- and time-dependent pattern, while that of NH_2_-QDs was partly dose-dependent and time-independent. Clearly, surface charge can affect the exocytosis of NPs, but there is no consensus on how charge affects the exocytosis of NPs.

Different surface groups make NPs enter different organelles in cells, thereby displaying different exocytosis patterns [32,99]. For example, the majority of PEG-modified PLGA NPs accumulated in lysosomes and underwent degradation, resulting in a long-term retention in cells, but B16-F10 cancer cell membrane coated PLGA NPs preferentially accumulated in the ER and Golgi apparatus, and then over 90% of internalized NPs was excreted within 4 h [32].

In summary, surface modification can affect the exocytosis process, but the effect of surface modification is also influenced by other factors like cell type.

### 5.3. Cell Type

Even though the exocytosis mechanism of NPs is still under debate, it is clear that cell type plays a non-negligible role [10,116]. Different cells may display different exocytosis behaviors when exposed to the same NPs. However, the influence is complex and variable, and no consistent conclusion has been achieved at present.

In a previous study of our group, we studied the exocytosis of 10 nm CDs-PEI in five cell lines with different proliferative capacities (rapidly proliferating cell lines: HeLa, A549, and BEAS-2B; slowly proliferating cell lines: A431 and MDA-MB-468). Within 24 h exocytosis, HeLa and BEAS-2B cells showed the lowest exocytosis capability, only about 45% of the total amount of CDs-PEI was expelled. In contrast, over 80% of CDs-PEI in MDA-MB-468 was excreted. A549 and A431 had similarly moderate efflux ratios (60%) [37]. The results suggested that the exocytosis of NPs is not closely related to the cell division rate.

It is interesting to compare the exocytosis of NPs in cancer cells and normal cells [9], though this is usually neglected. Liu et al. reported that normal lung cells (BEAS-2B) reached the maximum exocytosis within 4 h, where about 50% of preloaded CDs-PEI was excreted, but lung cancer cells (A549) took a longer time to reach the maximum exocytosis, with 60% being excreted at 24 h [37]. Chu et al. reported that 50 nm SiO_2_ NPs in normal human esophageal epithelial cells (NE083) had an extremely low efflux rate compared with that in human lung carcinoma cells H1299 [9]. Al-Hajaj et al. found that the exocytosis of cysteamine-modified QDs in liver cancer cells (Hep-G2) was significantly higher than that in normal kidney cells (HEK293) in the first 3 h of exocytosis, but the amount of QDs retained in the two types of cells (30–40%) became the same after 6 h [71]. In addition, Wang et al. reported that Au nanorods were retained in A549 cells and normal bronchial epithelial cells (16HBE) without excretion within 72 h; however, the content of Au nanorods in normal primary adult stem cells decreased by 60% from 48 h to 72 h [28]. Based on these results, we cannot infer that cancer cells can excrete more NPs than normal cells. Yanes et al. investigated the exocytosis rate of P-MSNs in a variety of cancer cells and reported the order of exocytosis efficiency from high to low was A549 cells (87%) > MDA-MB-231 (81%) > Panc-1 (75%) > MCF-7 (61%) > MDA-MB-435 (36%) > H-9 (4%) [10]. The exocytosis difference in these cells is huge, though they are all cancer cells. Further related studies are needed to understand more clearly and quantitatively the exocytosis behavior of NPs from cancer and normal cells.

Another issue of concern is whether there is the exocytosis of NPs in macrophages. Macrophages are the body’s first line of defense, serving as a primary cell population capable of interacting with foreign substances, including NPs. The exocytosis of NPs in macrophages has rarely been studied, possibly because macrophages are able to “destroy” substances within them. However, as shown in Figure 7a–c, Ho et al. found macrophage Raw264.7 generally had a weaker ability to excrete these Au@PEG NPs compared with non-macrophage cells (C166 and HeLa) [116]. In contrast, the exocytosis of some Au@PEG NPs, such as C6E-1, C12A-1, and C12A-2, was much more difficult in HeLa cells than in Raw264.7 cells. In addition, Oh et al. found that the exocytosis of spherical Au NPs was more difficult in M1/M2-polarized Raw264.7 than in 4T1 cells [68]; however, an opposite trend was observed on the rod-shaped Au NPs. Recently, Ho et al. reported that ~70% of methoxy-PEG-Au NPs exited HeLa and bEnd.3 cells, while only ~15% and ~40% of them departed from Raw264.7 and C166 cells, respectively [76]. Clearly, the exocytosis of NPs in macrophages exists, and the exocytosis capacity is influenced by factors such as surface modification and the shape of NPs.

It is reputed that the difference in NP transport between cell types is related to the level of membrane protein expression (e.g., P-glycoprotein) and cholesterol content [16,59,136]. Strobel et al. found the exocytosis of CeO_2_ NPs was significantly reduced when cholesterol on the cell membrane was consumed [58]. Similar results were observed by Sipos et al. [55]. There are known differences in the activity and expression of P-glycoprotein in different cells, suggesting that P-glycoprotein may be involved in the elimination of multiple NPs [50,137]. In addition, P-glycoproteins have been found within lipid raft membrane domains, and cholesterol as an important component of the lipid raft was established as one of the modulators of P-glycoprotein function, pointing out the ability of cells to eliminate NPs being, at least in part, dependent on them. Al-Hajaj et al. attributed the different exocytosis of QDs in Hep-G2 and HEK293 cells to the different expressions of P-glycoproteins in two cell lines [71], which were detected by using a P-glycoprotein inhibitor elacridar and a P-glycoproteins inducer rifampin.

### 5.4. Extracellular Conditions

A few studies report that serum in a culture medium facilitates the excretion of NPs [34,98,135]. Panyam et al. found that the exocytosis of PLGA NPs in human arterial vascular smooth muscle cells (VSMCs) was inhibited when serum was absent or depleted in the culture medium [34]. Furthermore, exocytosis could be activated when bovine serum albumin (the most abundant serum protein) and somatomedin (PDGF) were added to the culture medium without serum. These demonstrated that the exocytosis of PLGA NPs was influenced and even highly dependent on serum proteins, possibly because proteins in the medium were taken up by the cells, thereby interacting with existing pathways and leading to a greater exocytosis of NPs [138].

Ca^2+^ is another crucial extraneous factor enhancing the exocytosis of NPs. Different from these exocytosis inhibitors, the influx of Ca^2+^ into cells usually accelerates the efflux of NPs (Table 1) [10,21,55]. In an early study, Chen et al. found more Au NPs were expelled from HT-29 cells when there was a higher concentration of Ca^2+^ in the culture medium [21]. Later, Sipos et al. found that the influx of Ca^2+^ resulted in a 2-fold increase in the exocytosis rate of NPs in vesicles (mostly in lysosomes), and such an enhanced exocytosis did not happen when a chelate (BAPTA-AM) was used to bind Ca^2+^ in the medium [55]. However, the exocytosis of NPs in the cytoplasm cannot be regulated by adding Ca^2+^. This demonstrates that Ca^2+^ is a key factor to regulate lysosomal exocytosis. An increase in cytosolic Ca^2+^ promotes the fusion of lysosomes with the plasma membrane and thus accelerates the Ca-dependent recycling of vesicles, leading to the enhanced release of NPs. The fusion of the lysosomal membrane with the plasma membrane needs protein synaptotagmin VII [139], which is a transmembrane protein that upon binding Ca^2+^ undergoes a conformational change to bind to the SNARE complex on the plasma membrane, facilitating membrane fusion. Escrevente et al. identified SNARE family proteins Rab11a and Rab11b as regulators of the Ca^2+^-induced lysosome exocytosis [64].

Other components in the culture medium may also affect the release of intracellular molecules via different mechanisms. Nguyen et al. found that the addition of K⁺ in a medium promoted the release of vesicles from the cell into the extracellular space [140]. Ren et al. observed that Zn^2+^ could change the exocytosis dynamics [141]. This may be a mechanism to regulate the exocytosis of NPs in the future.

As it has been observed that the components of the culture medium affect the exocytosis of NPs, it is not easy to obtain accurate exocytosis information by just considering NPs and cells when their culture environment changes. In the meantime, adjusting the composition of the culture medium may be an effective method to regulate the efflux process of NPs.

Summarizing the results on the influencing factors of NP exocytosis, we can obtain some general rules, including that small NPs are easier to be excreted from cells than big ones, and serum and Ca^2+^ in the culture medium facilitate the excretion of NPs. Other influencing factors also affect the exocytosis of NPs, but these factors interact with each other, making the observed impacts complex.

## 6. Conclusions and Perspectives

In this review, we summarize the progress on the exocytosis of NPs. Despite the limited number of studies on this topic, current progress still forms a rough framework on the exocytosis of NPs, including exocytosis mechanisms/pathways, exocytosis kinetics, and influencing factors. Apparently, there are more problems found than solved. To help newcomers enter this important field, we also summarize the experimental methods and general considerations.

As to the exocytosis mechanism, several pathways have been identified; however, detailed information has to be worked out, such as how NPs (both naked and in vesicles) transport between organelles and out of cells, the key factors influencing the exocytosis of NPs and the related general rules, and whether the initial location of NPs during exocytosis plays a critical role in the exocytosis of NPs.

At present, the exocytosis of NPs is usually studied with NP-laden cells in an NP-free medium, in which the endocytosis of NPs is often negligible and ignored. As the exocytosis and endocytosis of NPs are two highly related opposite processes that almost always coexist, studying these two opposite processes simultaneously will provide valuable and accurate information. Certainly, this is a highly challenging task, requiring the development of new techniques.

Another challenging task is to consider the effect of protein corona. In a biological environment, NPs are almost always coated with proteins and other biomolecules. For any specific NPs, multiple exocytosis pathways likely coexist, but the major difference between different NPs is the partition among these pathways, which would be significantly affected by the protein corona of NPs. A failure to consider the effect of the protein corona might be the major reason for the inconsistent conclusions among different studies, both in the endocytosis and exocytosis of NPs. To make it more complicated, the formation of the protein corona is a highly dynamic process, and the protein corona of NPs will be changing all the time during cellular processes [112]. Compared to endocytosis, it is more difficult to investigate the protein corona during exocytosis, which also requires the development of new techniques.

In recent years, the development of new techniques with ever higher temporal and spatial resolution, such as single-cell ICP-MS [142], mass cytometry [143], and microfluidics combined with amperometric detection [144,145,146] has helped us detect NPs at the single-cell level, which may be used to study the exocytosis of NPs in the future. Apparently, more new techniques with high-resolution/detection limits are highly demanded.

It is of great importance and value to bring the cellular study to the animal level. However, the in vivo environment is much more complicated than and different from the in vitro cell culture systems, for example, the human blood flow system makes particles have a faster exchange rate with proteins than the closed system in culture dishes [75]. The experimental design of studies on exocytosis should take in vivo environments into account and find effective strategies to adjust the cellular uptake and exocytosis of NPs in vivo. This will greatly help to realize the long-term retention time of NP-based drug delivery systems in target cells at a high dosage in vivo, which is essential for the clinical translation of nanomedicines.

Full of challenges and rewards, the field of the exocytosis of NPs demands more cooperation from different multidisciplinary fields.

## Figures and Tables

**Table 1:** Commonly used concentrations and mechanisms of exocytosis inhibitors and accelerators.

Chemical		Concentration	Pathway	Function	Ref.
Inhibitor	NaN_3_/4 °C	--	--	Inhibiting cell membrane fluidity and energy-dependent transport	[54]
	Bafilomycin A1	0.1~0.5 μM	Lysosomal	Inhibitor of endosomal acidification and lysosomal maturation	[55,56]
	Chloroquine	100 μM	Lysosomal	pH buffering and inhibiting lysosomal enzymes	[57]
	LY294002	250 nM~1 mM	Lysosomal	Inhibiting PI3 kinase and lysosomal exocytosis	[10,37]
	Vacuolin-1	5 μM	Lysosomal	Inhibiting Ca^2+^-dependent lysosomal exocytosis	[37]
	Methyl-β-cyclodextrin (MβCD)	1~10 mM	Lysosomal	Cholesterol depletion	[58,59]
	Wortmannin	10~33 µM	Lysosomal	Preventing the transport of endosomes to lysosomes	[17,60]
	U1866A	2.5 µM	Lysosomal	Altering cholesterol accumulation and affecting different intracellular trafficking pathways	[10]
	Monensin	14~50 μM	Golgi	Blocking transportation from the Golgi apparatus to the cell membrane	[56,61]
	Exo1 (2-(4-fluorobenzoylamino)-benzoic acid methyl ester)	10~100 µM	Golgi	Inducing collapse of the Golgi apparatus	[10,62]
	Brefeldin A	35~90 μM	ER/Golgi	Inhibiting transport from the ER to the Golgi apparatus	[42,56]
	Nocodazole	15~33 μM	microtubule-associated transport	Inhibiting microtubule formation	[37,59]
	Cytochalasin D	5 µM	--	Disruption of actin polymerization	[37]
	Verapamil	10 µM~2 mM	--	P-glycoprotein inhibitor	[56,63]
	Fumitremorgin C	5 µM	--	MDR-associated protein inhibitor	[56]
Accelerator	Ionomycin	10 μM	Lysosomal	Accelerating exocytosis by transporting Ca^2+^ into the cells	[64]
	A23817	--	Lysosomal	Promoting Ca^2+^ influx and accelerating lysosomal associated efflux	[65]

**Table 2:** Summary of current studies on NP exocytosis and its influencing factors.

NP (Size, Shape, and Surface Group)	Cell Line	Pre-Culture Dose and Time	Exocytosis Period	Summary	Ref.
CdSe/ZnS QDs (4.6 nm, spherical, COOH-modification) (6.9 nm, spherical, NH_2_-modification)	HFF-1	2.5/7.5/15 nM, 4/24 h	6 h	Exocytosis of NH_2_-QDs was much easier than that of COOH-QDs. Exocytosis of COOH-QDs depended on pre-incubtaion time and dose but that of NH_2_-QDs did not depend on preincubation time.	[25]
CdSe/ZnS QDs (7.3 nm, spherical, 2-mercaptoethylamine)	HeLa	25 μg/mL, 6 h	6 h	The exocytosis was fast in the first 1 h and around 60% of internalized QDs remained undischarged at 6 h. The QDs were mainly excreted via lysosomal and ER/Golgi pathways.	[36]
CdSe/CdZnS QDs (8–10 nm, spherical, L-cysteine (Cys)/cysteamine (CA))	HEK293, Hep-G2	100 nM, 3 h	6 h	The efflux of QD-CA increased with time from around 20% after 1 h to ~60% after 6 h in HEK293, while it slightly increased from over 40% after 1 h to ~50% after 6 h in Hep-G2. The efflux of QD-CYS increased with time for both cell lines, but the efflux percentages were over 80% in Hep-G2 cells and 20% in HEK293 after 6 h. P-glycoprotein (P-gp) enhanced the efflux by regulation of the synthesis and metabolism of cholesterol.	[71]
CDs-PEI (10 nm, spherical, PEI)	HeLa, A549, A431, BEAS-2B, MDA-MB-468	10/20 μg/mL, 4 h	24 h	MDA-MB-468 cells had the greatest capacity for exocytosis (>80%), while HeLa and BEAS-2B cells had the least (45%). Exocytosis reached the maximum in BEAS-2B, MDA-MB-468, and HeLa cells within 6 h, while in A431 cells after 12 h and the exocytosis in A549 cells continuously within 24 h. The exocytosis was mainly via lysosomal and ER/Golgi pathways, and it was microtubule-dependent in all cell lines. The exocytosis percentages were independent of the initial CDs-PEI content, and the main initial location may not decide the main exocytosis route.	[37]
Si QDs (65 nm, spherical, allylamine)	HUVECs	50/100/200 μg/mL, 4 h	4 h	Intracellular Si QDs decreased with time and gradually reached a plateau value (around 4 h), while more than 40% of internalized Si QDs remained in the cells. Dissociation constant of complexes between Si QD aggregates and receptors in the endosome was found to be a determining factor for the exocytosis capacity of Si QDs.	[14]
DPA-QDs (8 nm, spherical, D-penicillamine)	HeLa	10 nM, 1.5 h	4 h	>50% of internalized DPA-QDs were excreted. Exocytosis had a half-life of 21 min and reached saturation after 2 h.	[13]
Au NPs (10/20/40 nm, spherical, anionic/cationic zwitterionic/PEG)	U937	150 μM, 6 h	48 h	The exocytosis was not significantly governed by the size. The cationic NPs always had the lowest exocytosis, while the PEG-NPs always had the highest exocytosis.	[123]
Au NPs (10 nm, spherical, poly(quaternary ammonium))	HT-29	0.005/0.5 mg/mL, 2 h	2 h	Extracellular Ca^2+^ promoted exocytosis.	[21]
Au NPs (15 nm, spherical, KATWLPPR peptide/KPRQPSLP peptide)	HUVECs	8 nM, 4 h	6 h	Around 40% of the NPs with similar charge and size but different functionality were excreted at 6 h. KATWLPPR peptide (able to bind to membrane receptor) coated NPs appeared to progressively exocytose, KPRQPSLP peptide (unable to bind to membrane receptor) coated NPs had a complex profile, i.e., about 10% of exocytosed NPs were re-uptaken. The NPs retained their colloidal stability after exocytosis.	[11]
Au NPs (2 nm, spherical, 5 different hydrophobicity surface groups)	MCF-7	200 nM, 3 h	6 h	The dynamic system provided higher exocytotic efficiency than the closed system. It was the chemical structure, not the hydrophobicity of surface groups, that dramatically affected the exocytosis amount and rate of NPs, and aromatic NPs had the highest exocytosis.	[12]
Au NPs (20 nm, spherical, 12 types of PEG with different hydrocarbyl end group)	Raw264.7, C166, HeLa	1 nM, 6 h	24 h	The exocytosis of NPs significantly depended on the cell types and the hydrocarbyl end group. The exocytosis of NPs from Raw264.7 cells was more difficult than in C166 cells.	[116]
Au NPs (25 nm, spherical, methoxy-PEG/dodecyl-PEG/PEG)	Kera-308, HeLa, C166, Raw 264.7, bEnd.3	1 nM, 10 h	24 h/48 h	Alkyl chains on PEG improved exocytosis in all cell types, with different absolute values in different cell types. Methoxy-loading NPs exited via conventional exocytosis pathways, but dodecyl-loading NPs exited Kera-308 cells predominantly via unconventional exocytosis, accompanied by the enhanced secretion of sub-100 nm exosomes. NP alkylation increased exocytosis percentages by up to ∼4.3-fold (15% to 65%) in Raw264.7 cells and yielded a maximum exocytosis of 87% in Kera-308 cells.	[76]
Au NPs (50 nm, spherical, cRGDfK-PEG/PEG)	U87, MCF-7	12 μg/mL, 4 h	0.5 h	The exocytosis was only observed in cRGDfK-PEG-AuNPs other than PEG-AuNPs and only in U87 cells other than MCF-7 cells. The exocytosis kinetics was tightly related with the recycling of the αvβ3 integrin.	[98]
Au NRs (55.6*13.3 nm, rod, bovine serum proteins)	A549, 16HBE, MSC	150 μM, 24 h	72 h	Both A549 and 16HBE cells were unable to exclude Au NRs within 72 h. Au NRs in MSC cells were excluded effectively after 24 h.	[28]
Au NPs, Au NRs (18/58*14/110*16 nm, spherical and rod, PEG)	Raw264.7, 4T1	150 μM, 6 h	48 h	Au NRs (110 × 16 nm) left the macrophages more efficiently than the other nanoparticles, regardless of the phenotype, but it remained longer in the tumor cells. Nanoparticles exit macrophages and tumor cells mainly via lysosomal exocytosis.	[68]
Au NPs, Au NRs (14/50/74/20*30/14*50/7*42 nm, spherical and rod, transferrin)	STO, HeLa, SNB 19,	0.02 nM, 6 h	8 h	The removal of the transferrin-coated NPs is linearly related to size. The fraction of rod-shaped NP exocytosis is higher than spherical-shaped nanostructures.	[75]
SiO_2_ NPs (50 nm, spherical)	H1299, NE083, NL20	10 μg/mL, 48 h	3 h	NP clusters in lysosomes are more easily excreted by the cells than single NPs in the cytoplasm. The excretion of NPs appears to be much slower in NE083 than that of H1299 and NL20 cells.	[9]
SiO_2_ NPs (60/180/370/600 nm, spherical)	Hep-G2	80 μg/mL, 12 h	12 h	The smaller NPs were more easily cleaned out and a longer pretreatment time resulted in a lower exocytosis percentage. Clearance of the particles during the first few hours was more prominent, with final exocytosis percentages of 63%, 67%, 58%, and 38% for NPs of 60, 180, 370, and 600 nm, respectively.	[119]
MSN (130 nm, spherical, phosphonate/PEI/ folate)	A549, MDA-MB 231, MCF-7, MDA-MB 435, PANC-1, H9	20 μg/mL, 2 h	6/24/48 h	Among the three MSNs, phosphonate-MSNs had the highest exocytosis percentage in A549 cells. Exocytosis efficiency of phosphonate-MSNs at 24 h: A549 (87%) > MDA-MB231 (81%) > PANC-1 (75%) > MCF-7 (61%) > MDA-MB 435 (36%) > H9 (4%). Phosphonate-MSNs were primarily excreted via the lysosomal pathway, but not the Golgi pathway. The shape and appearance of MSNs was unchanged after exocytosis.	[10]
MSN (133 nm, spherical, polydopamine)	HeLa	50 μg/mL, 12 h	14 h	The MSNs were excreted via the Golgi pathway. After 14 h, 62% of intracellular MSNs was exocytosed.	[62]
CeO_2_ NPs (18.8 nm, octahedral/spherical shape)	HMEC-1	1–100 µg/mL, 24 h	24 h	The exocytosis occurred mainly via the lysosomal and Golgi pathways and was membrane-cholesterol-dependent. The exocytosed NPs could be retaken up by cells.	[58]
Nanodiamond (FND) (100 nm, particle, oxygen-containing groups)	HeLa, 3T3-L1, 489-2.1	80 µg/mL, 4 h	8 days	Around 15% of internalized FND was excreted in HeLa and 489-2.1 cells after 6 days, while the exocytosis was up to 30% in 3T3-L1. Cell division led the dilution of intracellular FND.	[39]
Single-walled carbon nanotubes (SWCNTs) (90.3 nm, carboxyl and hydroxyl groups)	Raw264.7	10 µg/mL, 24 h	5/24 h	The P2X7R-mediated pathway is the predominant molecular mechanism. Exocytosis was through the lysosomal pathway and dependent on microtubules.	[115]
PS NPs (50/100 nm, spherical)	A549, BEAS-2B	20 μg/mL, 4 h	6 h	100 nm PS was more difficult to be excreted than 50 nm PS. 100 nm PS was difficult to be excreted from A549 cells. Lysosomes in A549 cells and actin and microtubules in BEAS-2B cells were involved in the exocytosis.	[124]
PLGA (97 nm, spherical, BSA)	VSMCs	100/200 μg/mL, 1/3 h	6 h	Exocytosis was inhibited in the serum-free medium. Exocytosis was affected by the preincubation dose and time.	[34]
PLGA (100 nm, spherical, octa-arginine (R8) peptide and L-cysteine)	Caco-2	200 μg/mL, 3 h	--	Exocytosis was through the Golgi secretory pathway and Cys was important for mediating Golgi transport. 25%R8/75%Cys NPs showed the most efficient exocytosis.	[99]
PAMAM (NH_2_-/OH-/COOH-modification)	MCF-7/ADR	20 µg/mL, 3 h	12 h	PAMAM-NH_2_ displayed the strongest exocytosis (~70% at 12 h), while PAMAM-OH the lowest exocytosis (20% at 12 h). Lysosomal and ER/Golgi pathways were involved in the exocytosis of PAMAM-NH_2_ and PAMAM-COOH but none were involved in that of PAMAM-OH.	[56]
Polysaccharide cationic NPs (60 nm, spherical)	16HBE14o-	25 µg/mL, 0.5 h	4 h	Cholesterol depletion totally blocked the exocytosis. Exocytosis reached the maximum value (~80%) after 1 h.	[16]

## Data Availability

Not applicable.
